# Occurrence of potentially zoonotic and cephalosporin resistant enteric bacteria among shelter dogs in the Central and South-Central Appalachia

**DOI:** 10.1186/s12917-021-03025-2

**Published:** 2021-09-25

**Authors:** Ashutosh Verma, Kimberly Carney, Marina Taylor, Kaitlyn Amsler, Joey Morgan, Karen Gruszynski, Erdal Erol, Craig Carter, Stephan Locke, Ashton Callipare, Devendra H. Shah

**Affiliations:** 1 College of Veterinary Medicine, Harrogate, USA; 2 Center for Infectious, Zoonotic and Vector-borne diseases, Harrogate, USA; 3 Center for Animal and Human Health in Appalachia, Lincoln Memorial University, 6965 Cumberland Gap Parkway, Harrogate, TN 37752 USA; 4 Veterinary Diagnostic Laboratory, University of Kentucky, Lexington, Kentucky USA; 5 Department of Veterinary Microbiology & Pathology, Washington State University College of Veterinary Medicine, Pullman, Washington, USA

**Keywords:** Shelter dogs, Central and South-Central Appalachia, Multi-drug resistance (MDR), Antimicrobial resistance (AMR)

## Abstract

**Background:**

Antimicrobial resistance and presence of zoonotic enteropathogens in shelter dogs pose a public health risk to shelter workers and potential adopters alike. In this study we investigated the prevalence of zoonotic bacterial pathogens and cephalosporin resistant (Cef^R^) enteric bacteria in the feces of apparently healthy shelter dogs in the Cumberland Gap Region (CGR) in the US states of Kentucky, Tennessee and Virginia.

**Results:**

Fecal samples of 59 dogs from 10 shelters in the CGR of Central and South-Central Appalachia were screened for the presence of *Campylobacter jejuni*, *Clostridium perfringens, Salmonella* and Cef^R^ enteric bacteria. *C. jejuni*, *C. perfringens* were detected by PCR based assays. Culture and PCR were used for *Salmonella* detection. Of 59 dogs, fecal samples from 14 (23.7%) and 8 (13.6%) dogs tested positive for *cpa* and *hipO* genes of *C. perfringens* and *C. jejuni,* respectively. *Salmonella* was not detected in any of the tested samples by PCR or culture. Cef^R^ enteric bacteria were isolated on MacConkey agar supplemented with ceftiofur followed by identification using MALDI-TOF. Fecal samples from 16 dogs (27.1%) yielded a total of 18 Cef^R^ enteric bacteria. Majority of Cef^R^ isolates (14/18, 77.8%) were *E. coli* followed by, one isolate each of *Enterococcus hirae*, *Acinetobacter baumannii*, *Acinetobacter pittii*, and *Pseudomonas aeruginosa*. Cef^R^ enteric bacteria were tested for resistance against 19- or 24-antibiotic panels using broth microdilution method. Seventeen (94.4%) Cef^R^ bacteria were resistant to more than one antimicrobial agent, and 14 (77.8%) displayed multidrug resistance (MDR).

**Conclusions:**

This study shows that shelter dogs within the CGR not only carry zoonotic bacterial pathogens, but also shed multidrug resistant enteric bacteria in their feces that may pose public health risks.

## Background

Cephalosporin-resistant enteropathogens are prevalent worldwide and are a formidable threat to both public and animal health as many exhibit multi-drug resistance (MDR) [1]. This is concerning because extended-spectrum cephalosporins are listed as key antibiotics for treatment of bacterial infections in both humans and animals [2, 3]. Companion animals can serve as a reservoir of anti-microbial resistant (AMR) bacteria that have an increased potential for zoonotic transmission due to their intimate contact with humans [4–7]. While previous research on companion animal reservoirs for AMR has focused on clinically ill animals [4, 8, 9], the literature on the fecal analysis of healthy dogs, especially shelter dog population in the United States is lacking [10]. The 2019-2020 APPA National Pet Owners Survey reported 19% dogs obtained from animal shelter/humane society [9, 11]. Thus, monitoring for the zoonotic pathogens and the AMR in shelter dogs is important for understanding the risk to the human population and the environment.

Population of dogs housed in animal shelters are at increased risk of carrying and spreading a variety of enteric pathogens to both animals and humans. Some of the common enteropathogens of dogs are also important public health pathogens. For instance, *Camylobacter jejuni* (*C. jejuni*) is a zoonotic pathogen frequently detected in symptomatic and asymptomatic dogs [12]. A few studies have reported that living with diarrheic pets is a risk factor for campylobacteriosis in humans [13–20]. A recent multi-laboratory survey in the United States showed that 1.3% symptomatic and 1.1% asymptomatic dogs shed *Salmonella* in their feces [21]. Similarly, toxin producing *Clostridium perfringens* (*cpa-*positive *C. perfringens*) is present in diarrheic and healthy dogs and is also considered as potential zoonotic pathogen [22].

Some factors that contribute to the introduction, persistence, and spread of enteric pathogens in animal shelters include high animal population densities, lack of proper veterinary care, stressful and unsanitary housing conditions, limited funding, adoption across state boundaries, and high animal turnover [23]. Cultural and socio-economic factors unique to the Appalachian Region further complicate these issues and put these animals at a risk of getting infected, becoming carriers, and transmitting diseases to both animals and humans. The objective of this study was to detect occurrence of zoonotic enteropathogens including *C. jejuni*, *Salmonella, cpa-*positive *C. perfringens* and cephalosporin resistant (Cef^R^) enteric bacteria in the feces of dogs (owner surrender, free-roaming, feral) housed in ten animal shelters across three US states within the Central and South-Central Appalachian Region.

## Results

### Population demographics

Animal attributes of shelter location, sex (male or female), estimated age, and fecal score were recorded at the time of sampling (Table 1). The dogs sampled in this study included 23 (39%) female, 36 (61%) male, and had a mean age of 2.2 years (range 2 months to 10 years), and a mean fecal score of 2.95 (range 1-6; Purina Fecal Scoring Scale). A fecal score of 1 indicates a hard and dry stool, 3 is normal, and a fecal score of 6 is indicative of a watery stool with no solid composition.

**Table 1:** Demographics of shelter dogs carrying Cef^R^ enteric bacteria

Sample ID	Shelter ID	Breed	Age (years)	Sex	Fecal Score	Bacterial isolates	MALDI scores
CR01	KR	Pit mix	2	f	6	*E. coli*	2.51
CR02	KR	Lab mix	2	m	4	*E. coli*	2.40
CR03	LC	Pitbull	1	m	2	*E. coli*	2.54
CR04	LC	Rottweiler mix	4	m	3	*E. coli*	2.49
CR05	LC	Australian shepherd mix	3	m	2	*E. coli*	2.33
CR06	UC	Collie mix	0.17	m	3	*E. coli*	2.56
CR07	UC	Collie mix	0.3	f	3	*E. coli*	2.49
CR08	SL	Chihuahua	5	m	3	*E. coli*	2.49
CR09	SL	Lab	1.5	m/n	3	*E. coli*	2.58
CR11	SL	Pitbull	3	m	5	*E. coli*	2.41
CR12	SL	Pit/Shepherd mix	1	m	3	*E. coli Enterococcus hirae*	2.52 2.32
CR13	SL	Hound	1	m	3	*E. coli*	2.33
CR14	JO	Beagle mix	3	f	2	*Acinetobacter baumannii*	2.32
CR15	JO	Collie	2	f	2	*Acinetobacter pittii* *Pseudomonas aeruginosa*	2.35 2.46
CR16	KW	Border Collie	0.42	m	6	*E. coli*	2.44
CR17	KW	Chihuahua mix	2	f	2	*E. coli*	2.51

### Occurrence of *C. jejuni*, *C. perfringens and Salmonella* in shelter dogs

**Fig. 1:**
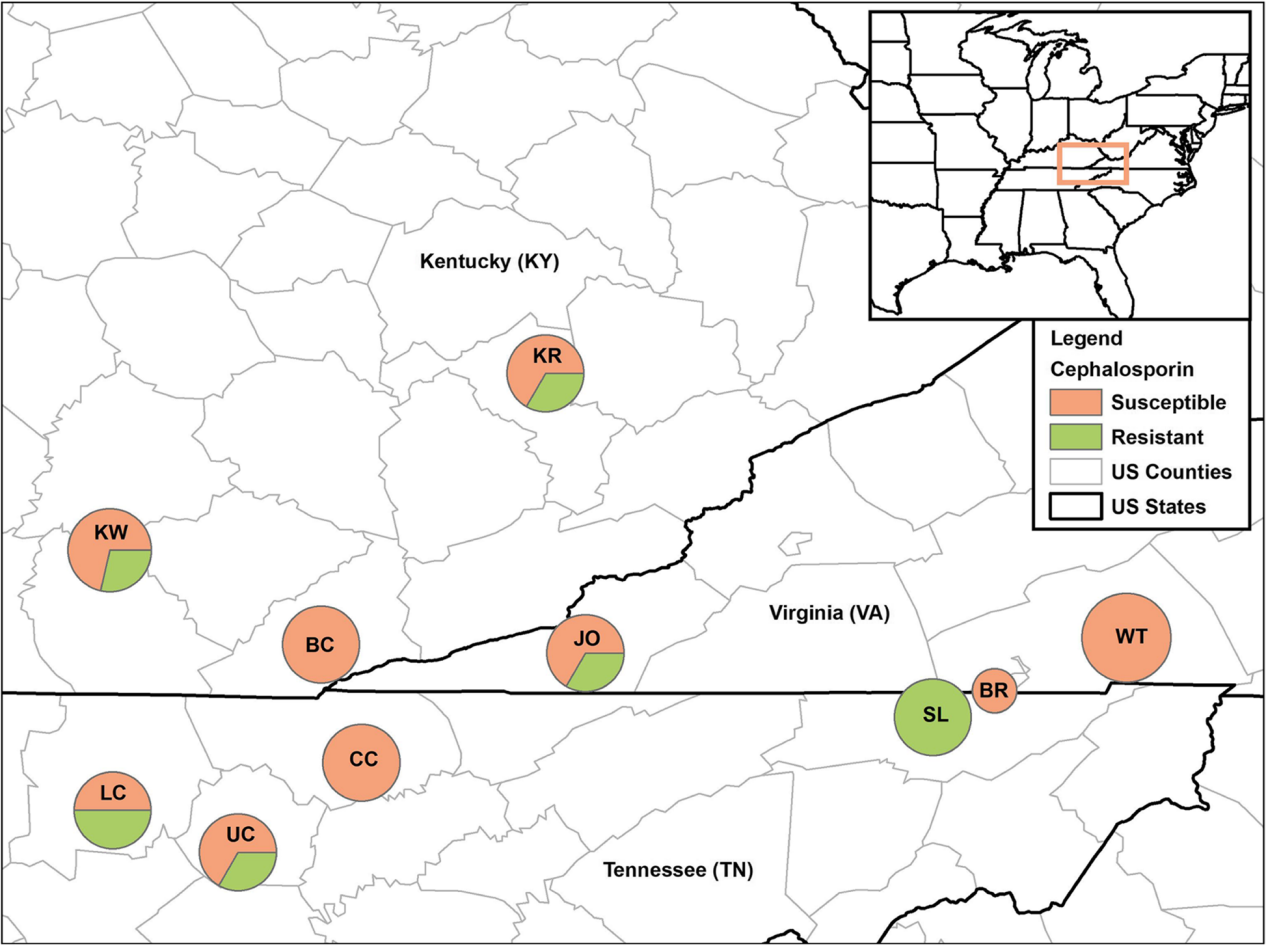
Cef^R^ enteric bacteria among shelter dogs. Map depicts the dog shelters in the US states of Kentucky, Tennessee, and Virginia. Fecal samples were collected from 8 dogs from Shelter WT, 7 dogs from Shelter KW, 6 dogs each from Shelters KR, BC, CC, LC, UC, SL, JO, and 2 dogs from Shelter BR. The proportions of Cef^R^ enteric bacteria within each shelter pie chart is shown. Map created with ArcMap 10.6 (Esri, Redlands, CA)

Of the 59 dogs tested in this study, 8 (13.6%; 95% CI: 6-30%) and 14 (23.7%; 95% CI: 13.6-36.6%) were positive for the *hipO* and *cpa* genes of *C. jejuni* and *C. perfringens*, respectively (Fig. 1). Three dogs were positive for both *C. jejuni* and *C. perfringens.* None of the dogs were positive for *Salmonella* by culture or by PCR methods. Out of 10 shelters, *C. jejuni* was detected in fecal samples collected from 3 (30%) shelters while *cpa*-positive *C. perfringens* was detected in fecal samples collected from 7 (70%) shelters. *C. jejuni* was detected most frequently in the fecal samples (4/8, 50%) collected from KR shelter. Many of the expected counts for chi-square tests were less than 5 due to the low number of samples and positive results except for *C. perfringens* and sex which was not statistically significant. Therefore, no statistical interpretation can be made regarding the other variables and test results for the sampled shelter dogs.

### Occurrence of Ceftiofur-resistant enteric bacteria

MALDI scores of Cef^R^ fecal isolates obtained in this study ranged from 2.32 to 2.56, allowing species-specific identification (Table 1). Cef^R^ resistant enteric bacteria were isolated from six (60%) out of the ten shelters sampled in this study. Of the 59 dogs sampled in this study, 16 (27.1%; 95% CI: 16.4-40.3%) tested positive for carriage of Cef^R^ bacteria. These 16 dogs yielded a total of 18 Cef^R^ resistant enteric bacteria (Table 1). A single/mono culture of Cef^R^ *E. coli* was isolated from 13 out of 16 dogs (81.2%)*.* The remaining three dogs (18.8%) yielded a mono culture of *Acinetobacter baumannii* (dog ID: CR14), mixed culture of Cef^R^ *E. coli and Enterococcus hirae* (dog ID: CR12) and a mixed culture of *Acinetobacter pitii* and *Pseudomonas aeruginosa* (dog ID: CR15)*.* Among the Cef^R^-positive dogs, the mean age was 1.9 years (range 2 months to 5 years), and the mean fecal score was 3 (range 2-6). Of note, 5 out of 6 (83.33%) samples from shelter SL demonstrated cephalosporin resistance, accounting for nearly 32% of all Cef^R^ bacteria isolated in this study. Only the variables sex and shelter when analyzed at the state-level had any statistical meaning due to small sample size and number of positives. State-level analysis showed a significant difference (*p*-value = 0.049) among the three states in terms of dogs having Cef^R^ enteric bacteria. At the state-level, Tennessee reported 45.8% (11/24; 95% CI: 25.6-67.2%), followed by Kentucky (4/19, 21.2%; 95% CI: 6.1-45.6%), and Virginia (2/16, 12.5%; 95% CI: 1.6-38.4%). Shelter SL is located within Tennessee which likely influenced the results.

### Occurrence of antimicrobial resistance

**Fig. 2:**
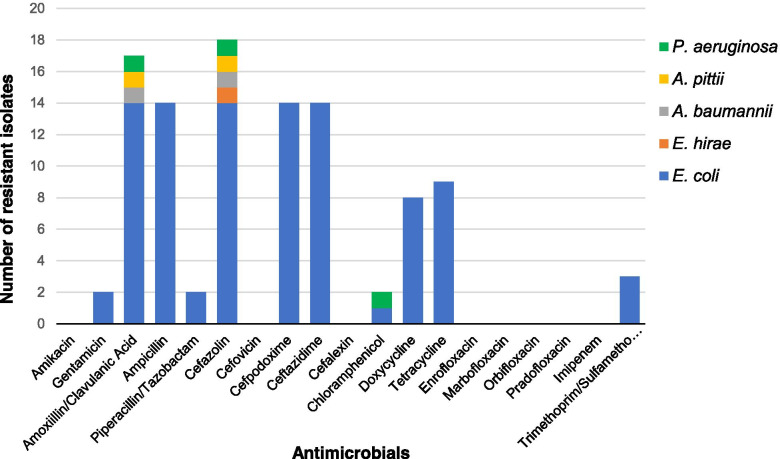
Antimicrobial resistance of bacterial isolates

Antimicrobial susceptibilities of Cef^R^ isolates (n=18) to a broad range of antibiotics that are relevant to companion animals were tested. Cef^R^ enteric bacteria isolated in this study also showed resistance to amoxicillin-clavulanate (n=17), ampicillin (n=14), cefazolin (n=18), cefpodoxime (n=14), ceftazidime (n=14), chloramphenicol (n=2), doxycycline (n=8), gentamicin (n=2), piperacillin (n=2), tetracycline (n=9), and trimethoprim/sulfamethoxazole (n=3) (Fig. 2).

**Fig. 3:**
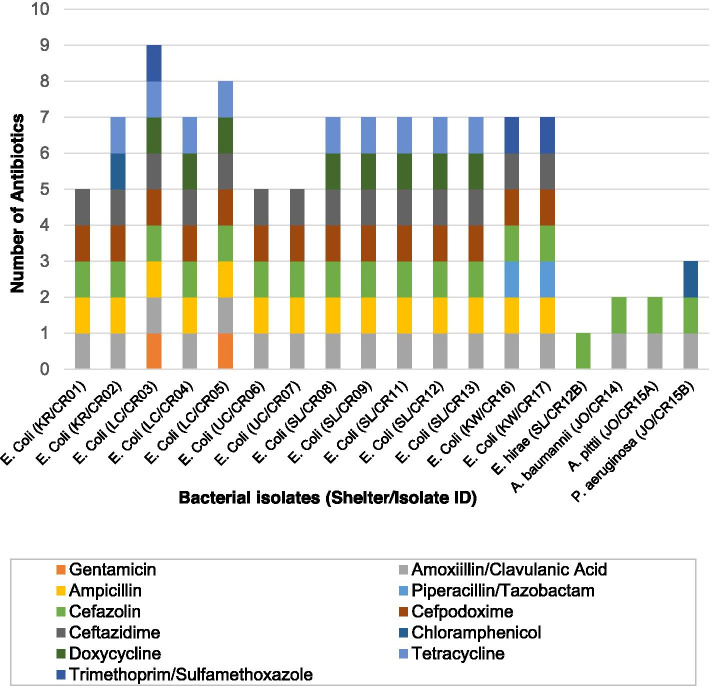
Multi-drug resistance patterns of individual bacterial isolates

Seventeen (94.4%) Cef^R^ isolates were resistant to more than one antibiotic (Fig. 3). Of the 14 *E. coli* isolates, 11 (78.6%) isolates exhibited MDR phenotype (resistance to three or more antibiotic classes). All *E. coli* isolates demonstrated resistance to amoxicillin/clavulanic acid, ampicillin, cefazolin, cefpodoxime, and ceftazidime. Eight out of 14 *E. coli* isolates were resistant to doxycycline; 9/14 to tetracycline; 3/14 to trimethoprim/sulfamethoxazole, 2/14 to gentamicin and piperacillin, and 1 to chloramphenicol (Fig. 3). One *E. coli* isolate (CR03) demonstrated resistance to 9 of the tested antimicrobials (Fig. 3). Isolates from different dogs within a same shelter had same antimicrobial resistance profiles, for example, isolates CR06 and CR07 (shelter UC); CR08, CR09, CR11, CR12, and CR13 (shelter SL); CR14 and CR15A (shelter JO); CR16 and CR17 (shelter KW) (Fig. 3).

## Discussion

Zoonotic and MDR enteric bacteria in shelter dogs pose a serious animal and public health concern. These dogs could serve as a reservoir of infections for other dogs, shelter worker or potential adopters. As noted earlier, some antimicrobials, such as extended spectrum cephalosporins, are listed as critical antibiotics for treating bacterial infections in both humans and animals [2, 3]. The occurrence of Cef^R^ enteric bacteria in shelter dogs in this study was 27.1% (16/59). These data corroborate with a recent study conducted in Ontario, Canada where average frequency of fecal carriage of Cef^R^ enteric bacteria in apparently healthy dogs was 26.5% [24].

The approach of initial selection for resistance to the third-generation cephalosporin (ceftiofur), has been used previously in cattle and poultry [25, 26]. Here, we employed similar approach to selectively isolate enteric bacteria that are not only resistant to cephalosporins, but also resistant to other antibiotic classes. For instance, 11 out of 14 *E. coli* isolates recovered in this study displayed MDR phenotype. The other common resistances noted in these isolates were against classes of antimicrobials that have been used for decades: penicillin, sulfonamide, aminoglycosides, and tetracycline. These findings are also consistent with those of Zhang et al (2018) in their study of fecal samples from dog parks and clinical settings.

A statistical association was found between the shelter of provenance and the presence of AMR in feces of the sampled dogs. While shelter characteristics were not recorded, the authors informally observed the shelters while collecting the samples. The shelters varied greatly in size, age of construction, training of personnel, stocking density, and overall perception of cleanliness. Interestingly, there were similar resistance profiles present within the population of individual shelters. For instance, *E. coli* isolates CR06 and CR07 in shelter UC, *E. coli* isolates CR08, CR09, CR11, CR12, and CR13 in shelter SL, *E. coli* isolates CR16 and CR17 in shelter KW, and *A. baumannii* and *A. pittii* isolates CR14 and CR15A in shelter JO had similar resistance profiles. These data suggest that dog-to-dog or point source transmission was likely occurring via environmental contamination or direct contact during socialization, even though the dogs were individually housed within each shelter. This provides an area for future investigation covering biosecurity, housing design, education, and disinfection processes to elucidate the horizontal transmission of resistant bacteria.

In addition, we found that 8 (13.5%) and 14 (23.7%) fecal samples tested in this study were positive for *C. jejuni* and *cpa-*positive *C. perfringens*. Both *C. jejuni* and *C. perfringens* are considered zoonotic pathogens [12, 22]. Although likely, it is currently unknown if any of these bacteria are transmitted from shelter dogs or environment to other dogs or people in proximity within these shelters. Other research groups have demonstrated the potential for transmission of different bacterial pathogens between dogs and humans. Recently, 78% of the S*taphylococcus* spp. isolated from healthy dogs were reported to exhibit multi-drug resistance [6, 27]. Guardabassi and colleagues reported that 46% of pet owners carried the same strain of *Staphylococcus pseudintermedius* as their dogs with deep pyoderma [6]. Similarly, other studies have also reported transmission of methilicin-resistant staphylococci from companion dogs to people in close proximity [28, 29]. Between 2016-2019, CDC reported two MDR *Campylobacter* outbreaks, which included almost 150 human cases, linked to contact with pet store puppies [30]. Although *Salmonella* was not detected in our study, zoonotic transmission of *Salmonella* from dogs to human has been reported [31–36]. The carriage and potential for transmission of zoonotic bacterial pathogens such as *Campylobacter* between clinically ill companion animals and humans is not a novel concept [19, 37]. However, this data shows that apparently healthy shelter dogs can be carriers of zoonotic and MDR enteric bacteria and may pose a hazard for pet owners, an increased occupational risk for animal care and veterinary staff, as well as a need for further research on this topic. Several bacterial species isolated in this study are known opportunistic human pathogens. For example, dog feces serve as a potential reservoir for *E. coli* with potential for extraintestinal infections such as urinary tract infections in humans [38–40]. The pathogen-AMR combinations detected in the current study have been identified as significant human pathogens under GLASS surveillance [41]. The results of interest include *E. coli* with resistance to penicillins, third and fourth generation cephalosporins, sulfonamides/trimethoprim. Although *Acinetobacter* spp. is on the GLASS surveillance list, the isolates in this study showed resistance only to penicillins and cephalosporins, which are not the antibiotic classes of interest. The ecology, epidemiology, and potential public health significance of the organisms in this specific situation of animal shelters is currently unknown and requires further investigation [42, 43].

One of the inherent limitations for this study is the lack of medical history for each dog. For example, the prior antimicrobial use of these dogs is unknown, as they were an undisclosed mix of captured and owner-surrendered dogs. It is worthwhile to note that exposure to multiple antimicrobials is not uncommon in many shelter situations [44] as upper respiratory (canine infectious respiratory disease-CIRD), gastrointestinal and heartworm diseases are frequently encountered in shelters. Broad-spectrum antibiotics such as doxycycline, may be used as part of treatment regimen for the above conditions [45]. Additional antibiotic treatments in shelters include amoxicillin-clavulanate, azithromycin, enrofloxacin, and trimethoprim-sulfonamide [46]. These antibiotics can select for multi-drug resistant bacteria within the shelter population, which may explain why resistance against all of these antibiotics were observed in the isolates in our study. It is possible that use of one or more antibiotics may have not only selected for MDR bacteria in the dogs screened in this study, but also leading to persistence and potential transmission between dogs.

## Conclusions

The results of this study show that shelter dog population can serve as a potential reservoir for zoonotic and MDR enteric bacteria and raise a possibility transmission to humans in proximity. Noting that many shelters allow the volunteers and general public to interact freely with these animals prior to adoption, the potential risk may not be just limited to the shelter staff. In case of vulnerable groups (geriatric, immunocompromised, undergoing chemotherapy), a screening protocol for zoonotic and AMR pathogens prior to adoption may be considered. Microbiome diagnostics may be of value in these situations and their usefulness is currently under investigation in our lab.

## Methods

### Ethics statement

This study was conducted on freely voided fecal samples collected under a protocol that was exempted by the Institutional Animal Care and Use Committee at the Lincoln Memorial University. Informed consent was obtained from the animal shelter directors to collect and use the fecal samples for research.

### Sample collection

**Fig. 4:**
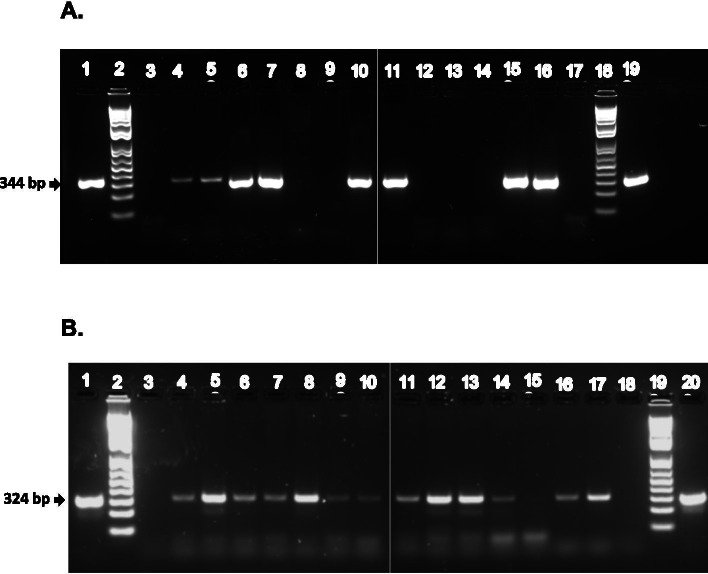
PCR identification of *C. jejuni* and *cpa-*positive *C.perfringens*. For *C. jejuni* identification **A**: Lanes 1 and 19, *C. jejuni* genomic DNA; lanes 2 and 18, 100bp DNA ladder; lanes 3 and 17, no template control; lanes 4, 5, 6, 7, 10, 11, 15 and 16, *C. jejuni hipO* positive samples; lanes 8, 9, 12, 13 and 14, *C. jejuni hipO* gene negative samples. For *C. perfringens* identification. Samples in lanes 1-10 and 11-19 were run on the top and bottom halves of the same gel, respectively. **B**: Lanes 1 and 20, *C. perfringens* genomic DNA; lanes 2 and 19, 100bp DNA ladder; lanes 3 and 18, no template control; lanes 4, 5, 6, 7, 8, 9, 10, 11, 12, 13 and 14, 15, 16 and 17, *C. perfringens cpa* gene positive samples. Lane 15 has a faint band. All samples were tested at least twice. Samples in lanes 1-10 and 11-20 were run on the top and bottom halves of the same gel, respectively

During Summer 2019, fecal samples were collected from 59 apparently healthy dogs, varying in sex and age, housed in 10 different shelters in the Appalachian Region of Kentucky, Tennessee, and Virginia (Fig. 4). The animals were apparently healthy, of varied ages and sexes at the time of sample collection. Fresh fecal samples (9-50 g) were collected from freshly voided samples in the individual kennel. Upon collection into plastic zip-top bags, samples were immediately placed on ice and transported to the lab for further processing. Demographic information including shelter location, animal age, breed, sex, and fecal score (Purina scale) were recorded for each dog (Table 1).

### *PCR screening for C. jejuni*, *C. perfringens and Salmonella*

Total genomic DNA was extracted from each fecal sample (n=59) using QIAamp DNA Stool Mini Kit following manufacturer’s instructions (Qiagen, Germantown, MD). Extracted DNA were quantified and screened for detection of *C. jejuni, C. perfringens* and *Salmonella* by PCR as follows. For the detection of *C. jejuni*, a PCR targeting *hipO* gene was used as described previously [47]. Each 25 μL PCR reaction contained 1.25 U FastStart Taq Polymerase (Roche Diagnostics), 1X PCR buffer (Applied Biosystems), 0.2 μM hipO-F (5’ GACTTCGTGCAGATATGGATGCTT), 0.2 μM hipO-R (5’ GCTATAACTATCCGAAGAAGCCATCA), 5 μL of fecal DNA, and thermal conditions as described previously [47]. For amplification of *cpa* gene from *C. perfringens*, each 25 μL PCR mixture consisted of 12.5 μL of DreamTaq PCR master mix (Thermo Fisher Scientific, CA, USA), 0.25 μM cpa1F primer (5′-GCTAATGTTACTGCCGTTGA-3′), 0.25 μM cpa1R primer (5′-CCTCTGATACATCGTGTAAA-3′), and 1 μL of fecal DNA. The PCR was performed following method described previously [48]. PCR product size for *C. perfringens* was 324 bp. For PCR detection of *Salmonella*, a well-conserved *Salmonella* gene *invA* was targeted, as described previously [48]. Briefly, each 25 μL reaction consisted of 0.04 μM invA_F primer (5′ GTGTCCTTTGGTATTAATCC-3′), 0.04 μM invA_R (5′-GTCTGAGCACTTCTTTAAG-3′) primer, 12.5 μL of DreamTaq PCR master mix (Thermo Fisher Scientific, CA, USA), and 2 μL of fecal DNA. The thermal conditions consisted of 95 °C for 5 min, followed by 39 cycles of 20 s at 95 °C, 20 s at 54 °C, 20 s at 72 °C, and a final for 5 min at 72 °C. PCR product size for *Salmonella* was 250 bp. Positive controls (genomic DNA extracted from a reference strain of each tested bacteria) and negative control (water) were included during each PCR run. PCR amplicons were analyzed by 1.5% agarose gel electrophoresis under standard conditions and stained by GelGreen Nucleic Acid Gel Stain (Biotium, Inc., Fremont, CA).

### Isolation of salmonella

For isolation of *Salmonella*, 1 g of fecal sample was resuspended in 10 ml of buffered peptone water (BPW) and incubated overnight at 37°C for 24 h. Subsequently, 10 drops of sample-BPW suspension were transferred to 10 ml of iodine-supplemented tetrathionate broth (TTB, Hardy Diagnostics) and incubated at 37°C for 24 h. Next, 10 μl of sample-TTB suspension was inoculated on XLT-4 agar (Hardy Diagnostics) plates and plates were incubated at 37°C for up to 24 h. Suspect *Salmonella* colonies were purified by replica plating and stored at −80°C in 15% (v/v) phosphate-buffered glycerol.

### Isolation of ceftiofur-resistant enteric bacteria

One gram of fecal sample was resuspended in 5 ml of phosphate buffered saline (PBS) and incubated at room temperature for 5 min. Fifty and 150 μl of sample were transferred onto a MacConkey agar plate supplemented with Ceftiofur (8 μg/ml) and evenly spread over the surface of the agar as described previously [25]. The plates were then incubated at 37°C for 15 to 18 h. Two to three colonies representing a unique colony morphotype were selected, transferred to 5 ml LB containing Ceftiofur (8 μg/ml), mixed well and then incubated again at 37°C for 15 to 18 h. Following incubation, the cultures were preserved in 15% (v/v) glycerol and frozen at -80°C. Approximately 1 μl of the sample was taken from the frozen stock, streaked onto a blood agar plate, and incubated at 37°C for 15 to 18 h. These plates were used for identification and characterization of isolates.

### Identification and characterization of isolates

The individual colonies from pure cultures were identified by MALDI-TOF (Bruker, Billerica, MA) following manufacturer’s instructions. The bacteria with scores above 2.0 were identified at the species level. The isolates from pure cultures underwent antimicrobial susceptibility testing by broth microdilution method using commercial plates (COMPGN1F and COMPGP1F, Trek Sensititer; ThermoFisher Scientific, Grand Island, NY, USA) in accordance to the guidelines established by the Clinical and Laboratory Standard Institute (CLSI, 2017). The concentrations (μg/ml) of the antimicrobials in COMPGN1F panel included Amikacin (4 – 32); Amoxicillin / clavulanic acid 2:1 ratio (0.25/0.12 – 8/4); Ampicillin (0.25 – 8); Cefazolin (1 – 32); Cefovecin (0.25 – 8); Cefpodoxime (1 – 8); Ceftazidime (4 – 16); Cephalexin (0.5 – 16); Chloramphenicol (2 – 32); Doxycycline (0.25 – 8); Enrofloxacin (0.12 – 4); Gentamicin (0.25 – 8); Imipenem (1 – 8); Marbofloxacin (0.12 – 4); Orbifloxacin (1 – 8); Piperacillin / tazobactam (8/4 – 64/4); Pradofloxacin (0.25 – 2); Tetracycline (4 – 16); Trimethoprim/sulfamethoxazole (0.5/9.5 – 4/76). The concentration of antimicrobials in COMPGP1F panel included Amikacin (16 – 32); Amoxicillin / clavulanic acid 2:1 ratio (0.25/0.12 – 8/4); Ampicillin (0.25 – 8); Cefazolin (2 – 4); Cefovecin (0.06 – 8); Cefpodoxime (2 – 8); Cephalothin (2 – 4); Chloramphenicol (8 – 32); Clindamycin (0.5 – 4); Doxycycline (0.12 – 0.5); Enrofloxacin (0.25 – 4); Erythromycin (0.25 – 4); Gentamicin (4 – 16); Imipenem (1 – 4); Marbofloxacin (1 – 4); Minocycline (0.5 – 2); Nitrofurantoin (16 – 64); Oxacillin+2%NaCl (0.25 – 2); Penicillin (0.06 – 8); Pradofloxacin (0.25 – 2); Rifampin (1 – 2); Tetracycline (0.25 – 1); Trimethoprim/sulfamethoxazole (2/38 – 4/76); Vancomycin (1 – 16). *S. aureus* ATCC 29213, *Streptococcus pneumoniae* ATCC 46619, *Enterococcus faecalis* ATCC 29212, or *Escherichia coli* ATCC 25922 were tested weekly as quality assurance to validate the test. The interpretation of antimicrobial susceptibilities was based upon the most recent CLSI guidelines as provided by the manufacturer. For *E. coli*, the isolate that were resistant to three or more antibiotic classes were labeled as MDR following the CDC-NARMS guidelines (https://www.cdc.gov/narms/resources/glossary.html). Because of their intrinsic antibiotic resistance, *Pseudomonas* spp. and *Acinetobacter* spp. were labeled MDR only if they were resistant to two or more of antibiotic classes for which they are not known to be intrinsically resistant (Shah et al., 2019).

### Statistical analysis

Statistical analysis was performed using SPSS 26 (IBM, New York). Briefly, test results for *C. perfringens*, *C. jejuni* and Cef^R^ enteric bacteria were compared to variables: shelter location, age, sex, and fecal score using chi-square test or Fisher’s exact test. Breed was not included in analysis due to too much variation in data collected. Additionally, shelter locations were grouped at state-level and dogs were categorized as < 1 year old or ≥ 1 year old to improve results of statistical analysis. A *p*-value of <0.05 was considered significant.

## Data Availability

The datasets used and/or analysed during the current study are available from the corresponding author on reasonable request.

## Abbreviations

AMR: Antimicrobial Resistance
Cef^R^: Cephalosporin Resistant
CDC: Centers for Disease Control and Prevention
CGR: Cumberland Gap Region
CLSI: Clinical and Laboratory Standard Institute
GLASS: Global Antimicrobial Resistance *Surveillance* System
MDR: Multi-Drug Resistant
MOUs: Memoranda of Understanding
WHO: World Health Organization
